# Physiological Antagonism

**Published:** 1896-08

**Authors:** Geo. E. Johnson

**Affiliations:** Fort Wayne, Ind.


### Physiological Antagonism.

BY DR. GEO. E. JOHNSON, FORT WAYNE, IND.

Read before the Indiana Dental Society, June 30, 1896.

By "Physiological Antagonism" in the science of medicine is meant a balance of opposed actions on particular organs or tissues.

I imagine I hear you say "he is not a little sugar-pill man or a benedict of Hahnemann" although I do acknowledge that some diseases are cured by contraries and some by similars, but similars are contraries so far as they remove the causes of disease or abnormal function, so the law of antagonism still holds sway.

"Similia simillbus curantur" operates in only a limited number of diseases, so we cannot rely on treating or duplicating symptoms for restoring lost equilibrium, or pathological physiology.

I cannot in the time allotted to a paper do more than treat the physical basis of the principle of antagonism and illustrate the mode of action and application of some of the remedies used in dental and oral diseases. The basis principle of an opposition of action finds its strongest support in the mechanism of functions. In the brain, medulla oblongata and cord are centers which control every function of the physical body, whether it be voluntary or involuntary.

In the great nervous system, viz.: the sensory, vaso-motor and the great sympathetic, we have a beautiful illustration of the division and union of labor, each having its office to perform, yet bound together in the bonds of sympathy making one grand system, working together for one high aim, the protection and preservation of the body of which they form a part. Opposing forces to maintain the equilibrium is demonstrated in the spasm-center of Northnagel, which is a center of extreme reflex sensibility situated in the medulla oblongata, and just above it, Setchenow's inhibitory center, with its correcting or restraining influence over reflex movements. If there were no antagonist or governor for the spasm-center, think of the very unpleasant reflex effects from every trifling peripheral irritation, but each is a force and when correctly balanced the nervous system sustains no unpleasant shock or results such as reflex neuralgia, lockjaw, or tetanic convulsions. We have an admirable illustration of opposing forces in the cardiac and respiratory mechanism producing order and rhythm. The movements of the large vessels are regulated by the largest of the cranial nerves, the pneumogastric (or parvagum) which in turn has its center in the medulla, and supplies the voice and respiration with motor and sensory filaments and the pharynx, œsophagus, stomach and heart with motor, and as the heart is also supplied from the recurrent laryngeal and great cardiac plexus of the sympathetic, you will readily observe the opposition of forces, and how the vascular tonus is maintained by the dilator and constrictor actions.

Perhaps the best illustration of opposing forces may be shown in the application of cold and heat in the following experiment: Lay the heart of a turtle or frog on a metallic plate, and if maintained at the normal temperature it will continue to act rhythmically for some time, but upon cooling the plate with ice you may slow or arrest its action; then, on applying heat it begins to pulsate again and still more rapidly as the heat is increased within proper limits. Another experiment: Drop a small quantity of serum, containing a slight trace of muscarine and the heart will be arrested diastole, but upon applying a 0.2 of atropia the pulsations begin again.

Reciprocity of action in the nervous mechanism regulates the blood pressure in the vascular system, and thus prevents fatal results. Should the arterioles of the body dilate from local or constitutional cause the heart increases its action automatically; on the other hand, should the arterioles contract or take on a spasm the heart's action is as suddenly lessened, thus maintaining the equilibrium and preventing breach or rupture of the vascular system.

The same reciprocity of action regulates the respiratory movements and many other functions we will not discuss in this paper as this will suffice to demonstrate the basis principle of opposing forces in their beneficence, and I shall now apply the principle in the treatment of inflammation, counter-irritation, tetanus, facial paralysis, hemorrhage, neuralgia and anæsthetics or anodynes.

Taking it for granted you undersand the pathology of the different stages of inflammation I shall treat it from the science of physiological therapeutics. Either local or constituional remedies, or both, should be applied or administered to antagonize the dilatation (or paresis) of the walls of the arteries and arterioles in the hyperæmic stage, but this treatment in stasis or exudation would be a very serious mistake. Aconite and iodine as local, and quinia and morphia as constitutional remedies, will raise the tonus of the arterioles, check the amœbiform movements of the leucocytes and the outward diffusion of albumen, fibrine and salts, but chloral hydrate is especially useful prior to the stage of complete stasis, as it diminishes the heat, dissolves exudations, and has a hypnotic action to quiet restlessness.

Local applications of heat at first produces hyperæmia by the dilatation of the arterioles (paresis of the sympathetic filaments), but continued application of heat stimulates the vaso-motor to increased nutrition and thereby removes the difficulty if the heat does not greatly exceed the normal temperature of the body. While, on the other hand, cold first produces contraction or spasm of the vaso-motor filaments (anæmia) and if remitted will result in hyperæmia, and if extreme cold is long continued will result in stasis and finally gangrene by impoverishing the parts.

Counter-irritation circumscribes itself by vesication, induces an afflux of blood, stimulates the filaments of the vaso-motor by paralyzing the end filaments of the sympathetic. Tetanic convulsions proceed from peripheral irritation, a wound or a concussion, and you ask an explanation. This is an abnormal excitability or irritability of Nothnagal's spasm-center due to the functional inactivity of the inhibitory center of Setchnow.

What are the symptoms and how antagonized? A sudden deathly pallor which means contraction or spasm of the arterioles of the brain, next a partial or complete paresis of the pneumogastric. What is the result? Suspension of respiration, and next cyanosis, as a result of suspension of respiration, and lastly paresis of the heart.

We now understand the exact pathological condition along the line from cause to effect. How antagonize? Relieve the spasm at the starting point and the symptoms will quickly disappear. Inhalation of nitrate of amyl, administration of chloral, bromide of potassium, physostigma and gelsemium are indicated, the same remedies being indicated in cases of poisoning by strychnia, as this produces the same functional disturbance.

The reverse, viz.: Facial paralysis, strychnia and electricity are indicated. Who in this age of medical science need to resort to the medley of ancient astringents, brown paper, spider webs or pow wow, for hemorrhage or hemorrhagic diathesis? Relaxation of the vessel walls and arterioles, and consequently acceleration of the heart's action are the conditions to antagonize, and the best remedies are ergot, digitalis, bromide of potassium, veratrum viride, etc., with tincture of chloride of iron.

We now come to the most interesting part of my paper. To very many neuralgias and anæsthetics, which I shall treat as pain and anodynes. Dr. Gross said: "If America had contributed nothing more to the stock of human happiness than anæsthetics the world would owe her an everlasting debt of gratitude."

But to analyze pain, what is it? how prevent it? or how antagonize it? Pain is composed of several elements and degrees of intensity, and is the result of real or imaginary injury.

I shall not treat from a psychological point of view but suffice it to say it is a consciousness or realization of an injury, real or imaginary, reflected to and through the spasm-center of Nothnagel exalting or exaggerating this center to extreme sensibility. Hence these two great forces of the animal economy, inhibition and spasm are thrown off their equilibrium by overpowering the inhibitory center, therefore, it is apparent that with the power of exciting inhibition, or suspending reflex action will prevent or relieve pain, or restore lost equilibrium, and this may be accomplished by two methods, local and general anæsthesia.

Any remedy which applied locally paralyses the end filaments of the sensory, or destroys the transmission of sensation to the center, prevents pain. This action we have in aconite, chloral, canabis India, cocaine and many other drugs varying in effectiveness.

Any remedy, or combination of remedies, which suspends reflex sensibility of the center without impairing other functions, is the agent we have been searching many years to find.

